# Supplementary material for: Proteogenomic characterization of MiT family translocation renal cell carcinoma
Source: Nat Commun. 2022 Dec 5;13:7494. doi: 10.1038/s41467-022-34460-w (PMC9722939; doi:10.1038/s41467-022-34460-w)
Supplement: Supplementary file 1 — Supplementary Information [file 41467_2022_34460_MOESM1_ESM.pdf]

Supplementary Information

Proteogenomic Characterization of MiT Family Translocation Renal Cell Carcinoma

Yuanyuan Qu, Xiaohui Wu, Aihetaimujiang Anwaier, Jinwen Feng, Wenhao Xu, Xiaoru Pei, Yu Zhu, Yang Liu, Lin Bai, Guojian Yang, Xi Tian, Jiaqi Su, Guo-Hai Shi, Da-Long Cao, Fujiang Xu, Yue Wang, Hua-Lei Gan, Shujuan Ni, Meng-Hong Sun, Jian-Yuan Zhao, Hailiang Zhang, Dingwei Ye, Chen Ding

Supplementary Figure 1

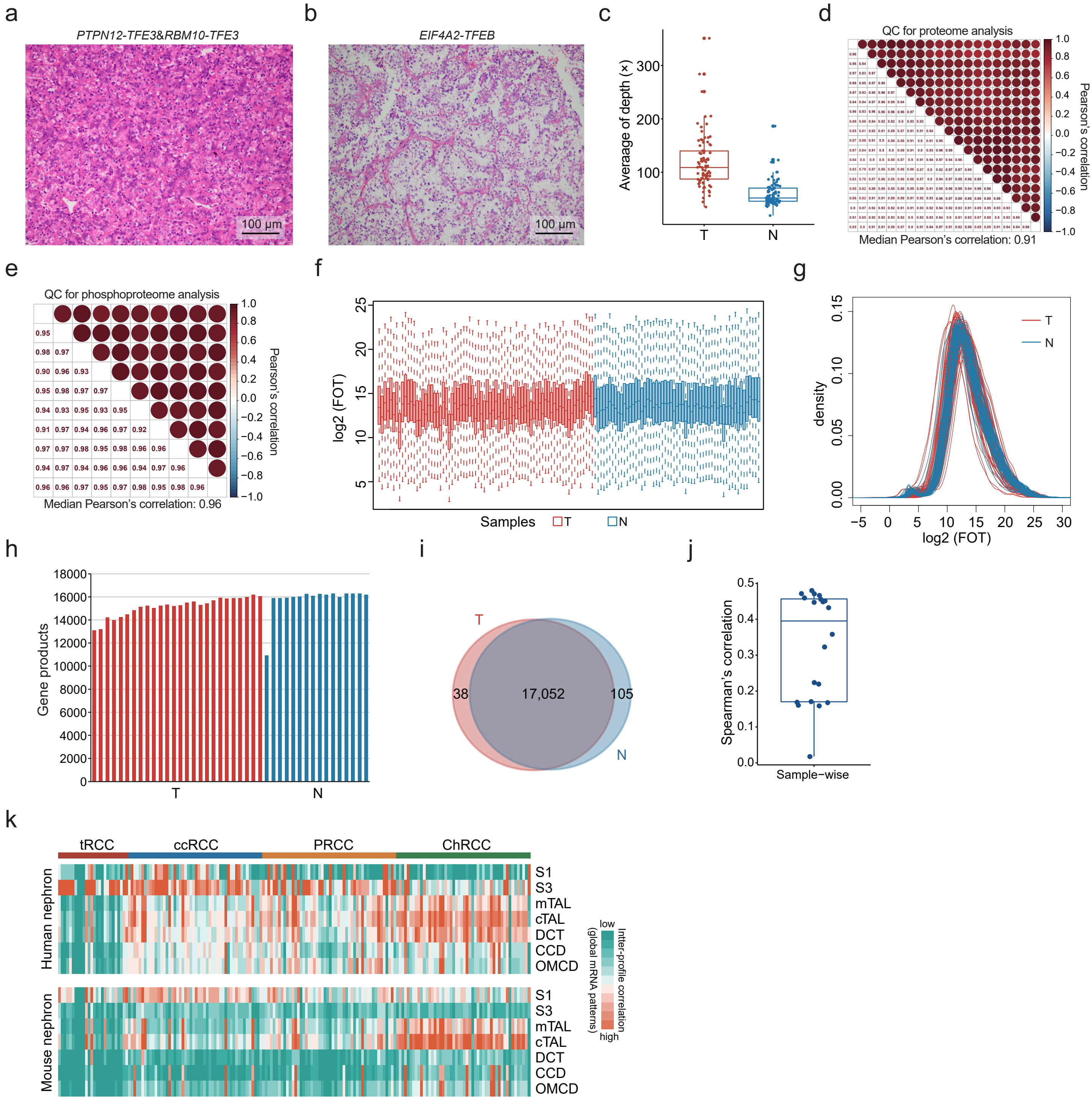

Supplementary Figure 1. Multi-omics Landscape of tRCC samples

a, Representative images of *PTPN12-TFE3&RBM10-TFE3* tRCC cases. The *PTPN12-TFE3&RBM10-TFE3* tRCC tumor cells were nest-like or glandular distribution with intermediate in size, abundant cytoplasm and indistinct cell boundaries, round nuclei with inconspicuous nucleoli. Scale bars: 100 μm. **b**, Representative images of *PTPN12-TFE3&RBM10-TFE3* tRCC cases. The *EIF4A2-TFEB* tRCC case had a solid nest pattern or papillary pattern resembling clear cell renal cell carcinoma. Scale bars: 100 μm. **c,** Coverage of tumor (n = 84) and NAT (n = 84) WES data. Boxplot show the median (central line), the 25–75% IQR (box limits), the ±1.5×IQR (whiskers). **d-e,** Longitudinal quality control of mass spectrometry for proteome and phosphoproteome analysis. **f**, Boxplot of the log2 (FOT) protein abundances for the tRCC tumor and NAT samples. Red indicates tumors, blue indicates NATs. Boxplots show the median (central line), the 25–75% IQR (box limits), the ±1.5×IQR (whiskers). **g,** Distribution of protein abundances in tumors (red) and NATs (blue) by a density plot. **h,** Bar plots showing the number of identified transcripts in tRCC tumor and NAT samples. **i,** The Venn diagram showing the overlap of transcripts in tRCC tumor and NAT samples. **j**, Boxplot showing the sample-wise mRNA-protein Spearman's correlation (n = 20). Boxplot show the median (central line), the 25–75% IQR (box limits), the ±1.5×IQR (whiskers). **k**, Heatmap showing inter-sample correlations (Pearson's correlation) between expression profiles of kidney malignancies (including tRCC, ccRCC, PRCC and ChRCC) and expression profiles of kidney nephron sites. S1 and S3, initial and terminal portions of the proximal tubule; mTAL, medullary thick ascending limb of Henle's loop; cTAL, cortical thick ascending limb of Henle's loop; DCT, distal convoluted tubule; CCD, cortical collecting duct; OMCD, outer medullary collecting duct.

Supplementary Figure 2

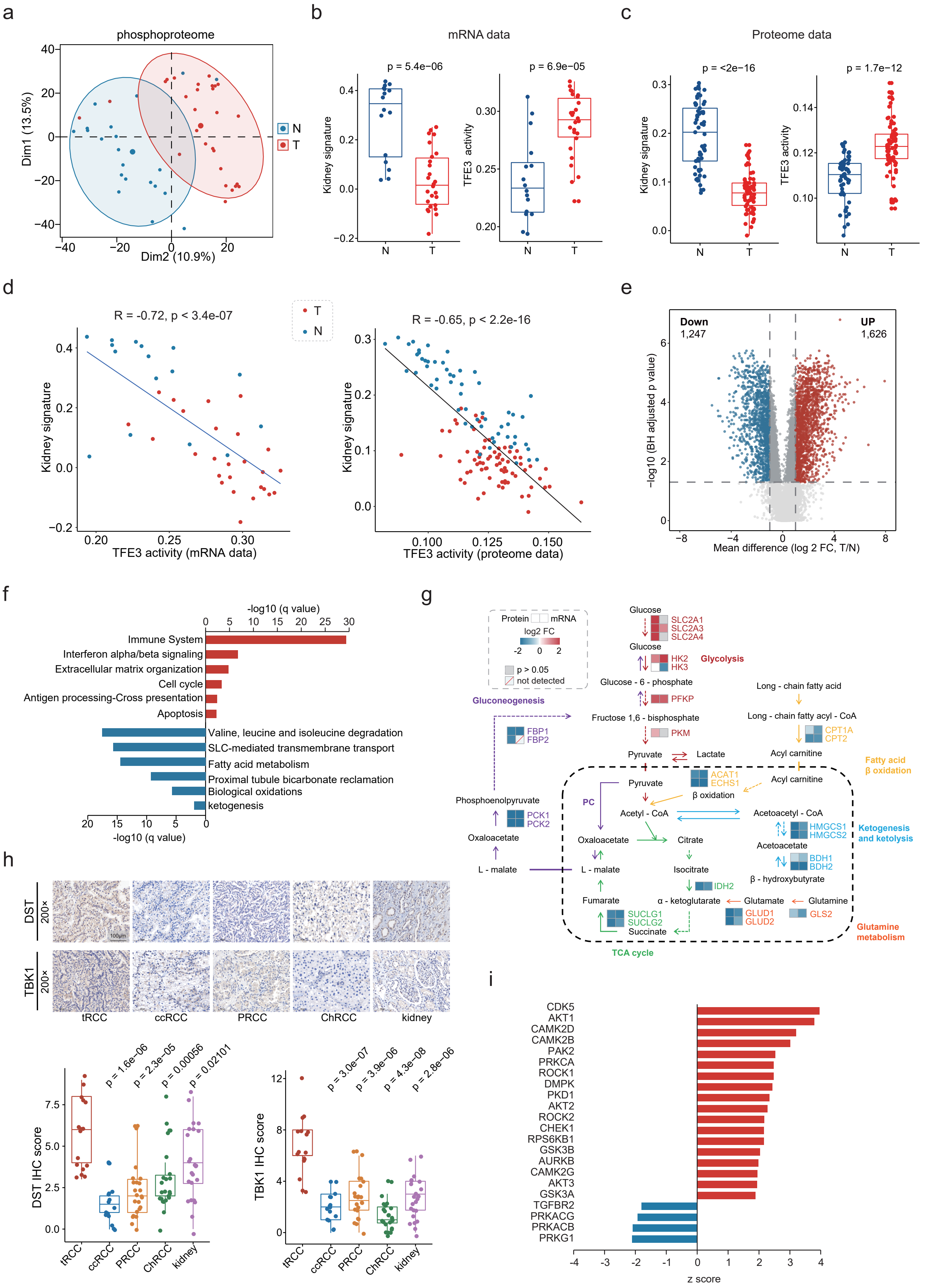

Supplementary Figure 2. Molecular Alterations in tRCC Tumors Compared to Adjacent Tissues

a, Global phosphoproteomics PCA plot. Red, tumors; Blue, NATs. **b-c,** Comparisons of mRNA (N, n = 16; T, n = 26) and proteome (N, n = 57; T, n =74) data-based kidney signature scores and inferred TFE3 activities between tumors and NATs. **d,** Kidney signatures scores were negatively correlated with inferred TFE3 activities in both mRNA and proteome data (two-sided Spearman's correlation test). **e,** Volcano plot showing differential expressed genes (two-sided Wilcoxon rank-sum test, B-H adjusted p value < 0.05, FC > 2) of tRCC tumor and NAT transcriptome. **f,** Differential expressed genes enriched biological pathways. **g,** Alterations of kidney metabolic pathways in tRCC tumors compared with NATs at transcriptome and proteome level. **h,** Representative IHC staining for DST and TBK1 in tRCC, ccRCC, PRCC, ChRCC, and normal kidney tissue. Scale bars: 100 μm. Boxplots showing the IHC scores of DST and TBK1 for tRCC (n = 16), ccRCC (n = 16), PRCC (n = 24), ChRCC (n = 24), and normal kidney tissue (n = 24) samples. **i,** KSEA analyses of kinase activities in tumors and NATs. Boxplots in panels **b**, **c**, **h** show the median (central line), the 25–75% IQR (box limits), the ±1.5×IQR (whiskers). P values are derived from two-sided Wilcoxon rank-sum test.

Supplementary Figure 3

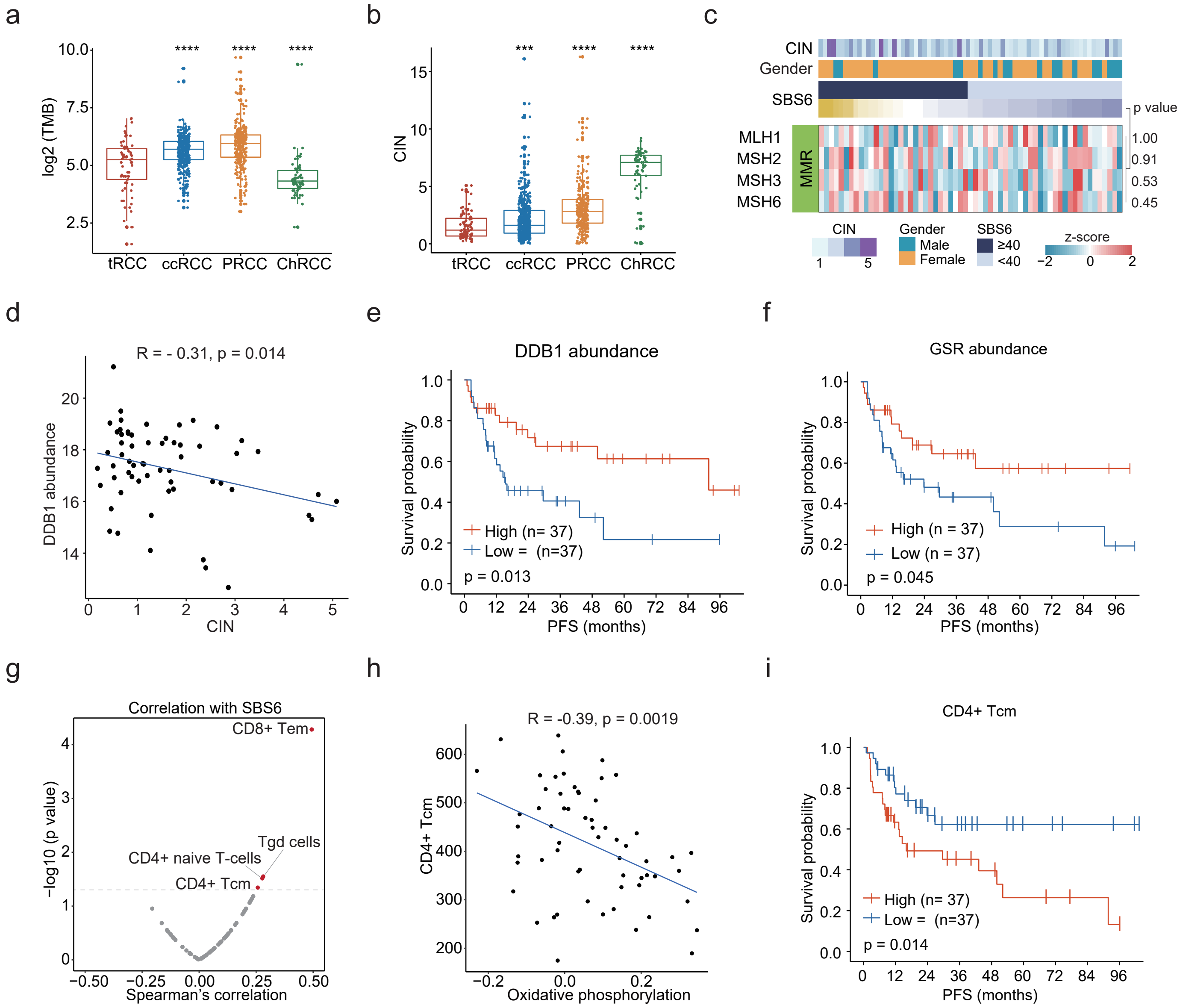

Supplementary Figure 3. Mutational Signatures SBS6 Associated Signaling Pathways

a-b, Comparison of TMB and CIN among different RCC subtypes (tRCC, n = 84; ccRCC, n = 529; PRCC, n = 289; ChRCC, n = 66). ccRCC, clear cell renal cell carcinoma; PRCC, papillary renal cell carcinoma; ChRCC, chromophobe renal cell carcinoma. Boxplots show the median (central line), the 25–75% IQR (box limits), the ±1.5×IQR (whiskers). P values are derived from two-sided Student's t test. *** p< 0.001, **** p < 0.0001. **c,** The expression level of mismatch repair proteins in tumors with and without SBS6 signature. P values are derived from two-sided Wilcoxon rank-sum test. **d,** Correlation between DDB1 abundances and CIN (two-sided Spearman's correlation test). **e,** Kaplan–Meier curves of PFS for patients with different DDB1 abundances (two-sided log-rank test). **f,** Kaplan–Meier curves of PFS for patients with different GSR abundances (two-sided log-rank test). **g,** Scatter plot showing the correlation of SBS6 and xCell immune signatures (two-sided Spearman's correlation test). **h,** Correlation between OXPHOS scores and CD4^{+} Tcm signatures (two-sided Spearman's correlation test). **i,** Kaplan–Meier curves of PFS for patients with different CD4^{+} Tcm scores (two-sided log-rank test).

Supplementary Figure 4

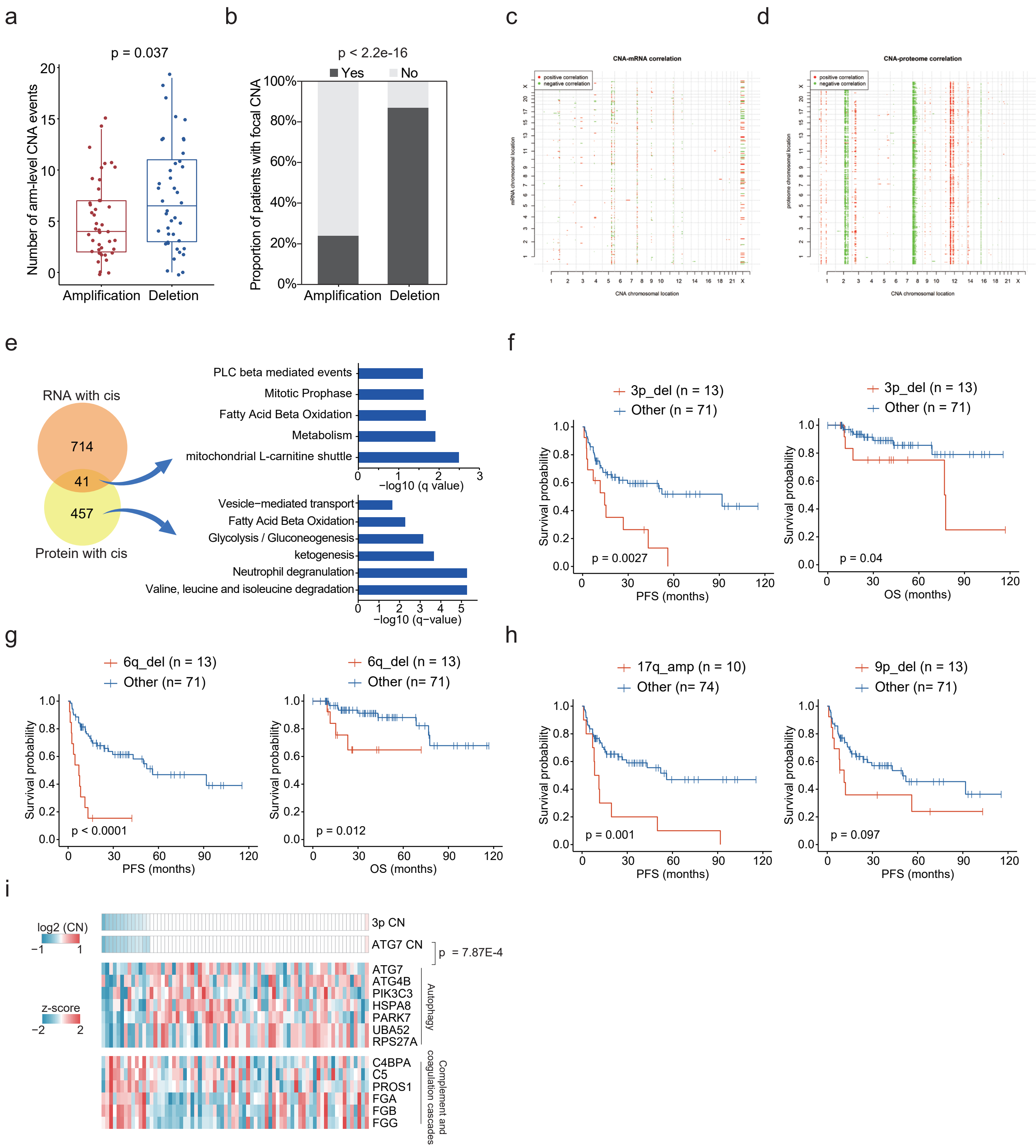

Supplementary Figure 4. Somatic Copy Number Alterations in tRCC Cohort

a, Comparison of arm-level amplification and deletion events in tRCC cohort (n = 42). Boxplots show the median (central line), the 25–75% IQR (box limits), the ±1.5×IQR (whiskers). P value is derived from two-sided Student's t test. **b,** Comparison of focal amplification and deletion events in tRCC cohort (Fisher's exact test). **c-d,** Effects of copy-number alternations on mRNA and protein abundance. Significant positive and negative correlations (two-sided Spearman's correlation test, FDR < 0.10) are indicated by red and green. **e**, *Cis*-effect at mRNA and protein levels and functional enrichment. **f,** Kaplan–Meier curves of PFS and OS for patients with and without 3p deletion (two-sided log-rank test). **g,** Kaplan–Meier curves of PFS and OS for patients with and without 6q deletion (two-sided log-rank test). **h,** Kaplan–Meier curves of PFS for patients with and without 17q amplification or 9p deletion (two-sided log-rank test). **i,** *Cis*-effect of 3p deletion on ATG7 protein abundance and *trans*-effects of 3p deletion on autophagy and complement cascade (two-sided Spearman's correlation test).

Supplementary Figure 5

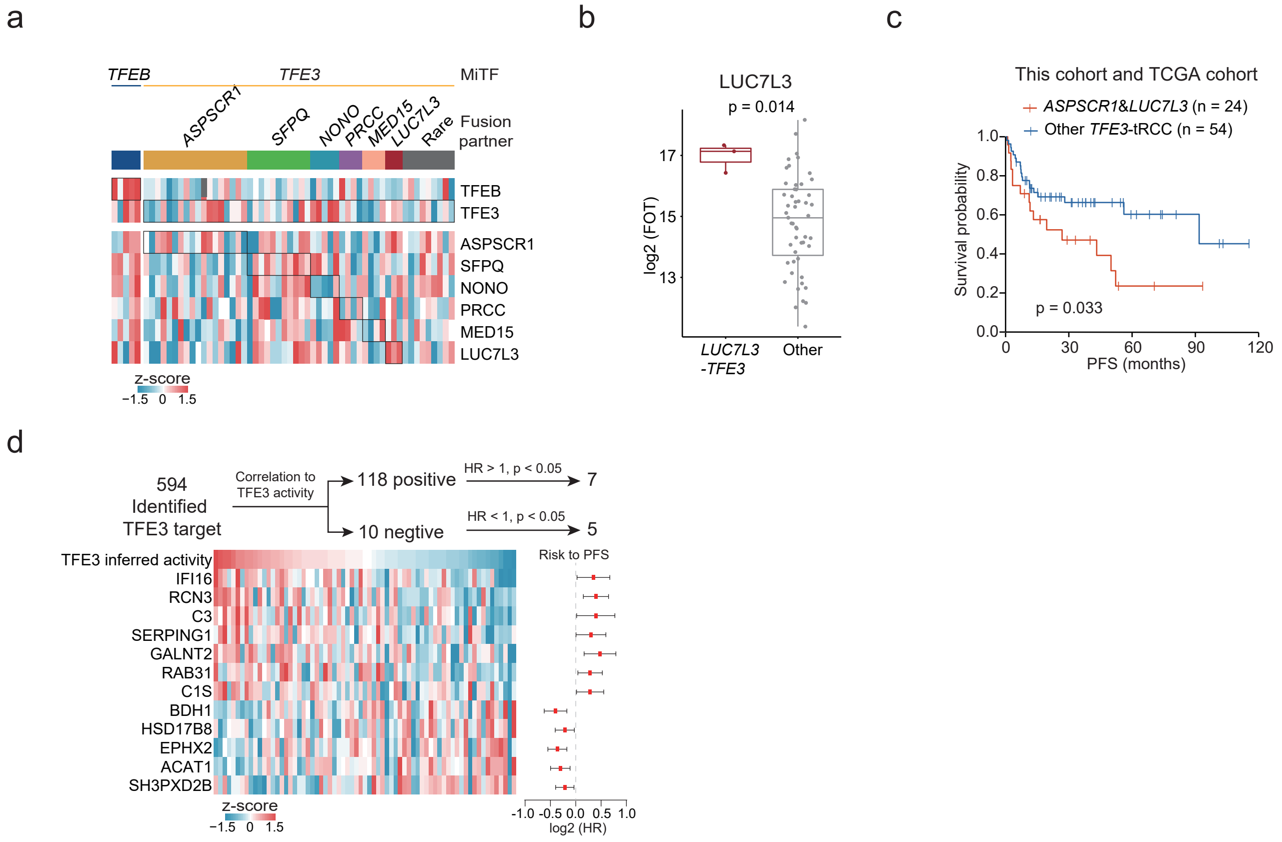

Supplementary Figure 5. Molecular heterogeneity of different fusion types of tRCC

a, Heatmap showing the protein abundances of TFEB, TFE3, ASPSCR1, SFPQ, NONO, PRCC, MED15, and LUC7L3. **b,** Comparisons of LUC7L3 abundances between *LUC7L3-TFE3* tumors (n = 3) and other tRCC tumors (n = 51). Boxplots show the median (central line), the 25–75% IQR (box limits), the ±1.5×IQR (whiskers). P values are derived from two-sided Wilcoxon rank-sum test. **c,** Kaplan–Meier curves of PFS for *ASPSCR1*&*LUC7L3 TFE3*-tRCC tumors versus other *TFE3*-tRCC tumors combining this cohort and the TCGA cohort (two-sided log-rank test). **d,** Screening of TFE3 targets associated with prognosis. Left, protein abundance. Right, HR for PFS of each protein, error bars indicate 95% confidence interval for HR.

Supplementary Figure 6

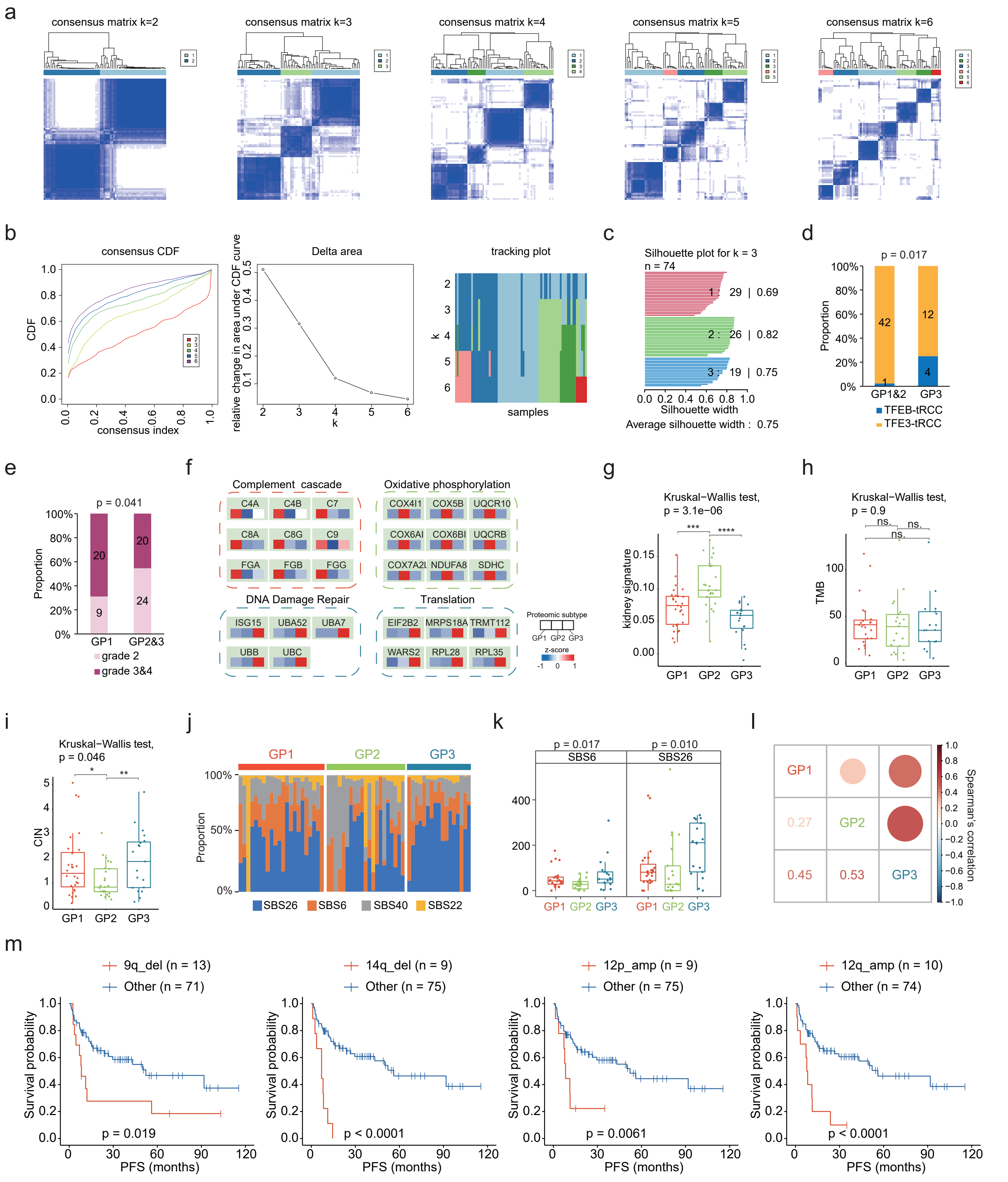

Supplementary Figure 6. Proteomic Subtyping of tRCC and Related Genomic Alterations.

a, Consensus matrices of the tRCC tumors from k = 2 to k = 6. Consensus clustering was conducted for the top 50% most-variant tumor and NAT DEPs. **b,** Cumulative distribution function plot, delta plot, and tracking plot corresponding to consensus matrices from k = 2 to k = 6. **c,** Silhouette plot (k = 3). **d,** The proportion of *TFE3*/*TFEB*-tRCC in GP3 and GP1&2 cases (One-sided Fisher's exact test). TFE3-tRCC-GP1&2, n = 42; TFEB-tRCC-GP1&2, n = 1; TFE3-tRCC-GP3, n = 12; TFEB-tRCC-GP3, n = 4. **e**, The proportion of grade 2 in GP3 and GP1&2 cases (One-sided Fisher's exact test). Grade 2-GP1, n = 9; Grade 3&4-GP1, n = 20; Grade 2-GP2&3, n = 24; Grade 3&4-GP2&3, n = 20. **f**, Signature proteins among three proteomic subtypes. **g-i,** Comparisons of kidney signature scores, TMB, and CIN among three proteomic subtypes (GP1, n = 29; GP2, n = 26; GP3, n = 19). P values are derived from two-sided Wilcoxon rank-sum test. **j,** Relative percentage of each mutation signatures in the three proteomic subtypes. **k,** Comparison of SBS6 and SBS26 among three proteomic subtypes (GP1, n = 29; GP2, n = 26; GP3, n = 19). P values are derived from Kruskal-Wallis test. **l,** Correlation plot of the arm-level CNA frequencies among three proteomic subtypes. **m,** Kaplan–Meier curves of PFS for patients with and without arm-level CNA events which was enriched in GP1 (two-sided log-rank test). Boxplots in panels **g-i**, **k** show the median (central line), the 25–75% IQR (box limits), the ±1.5×IQR (whiskers).

Supplementary Figure 7

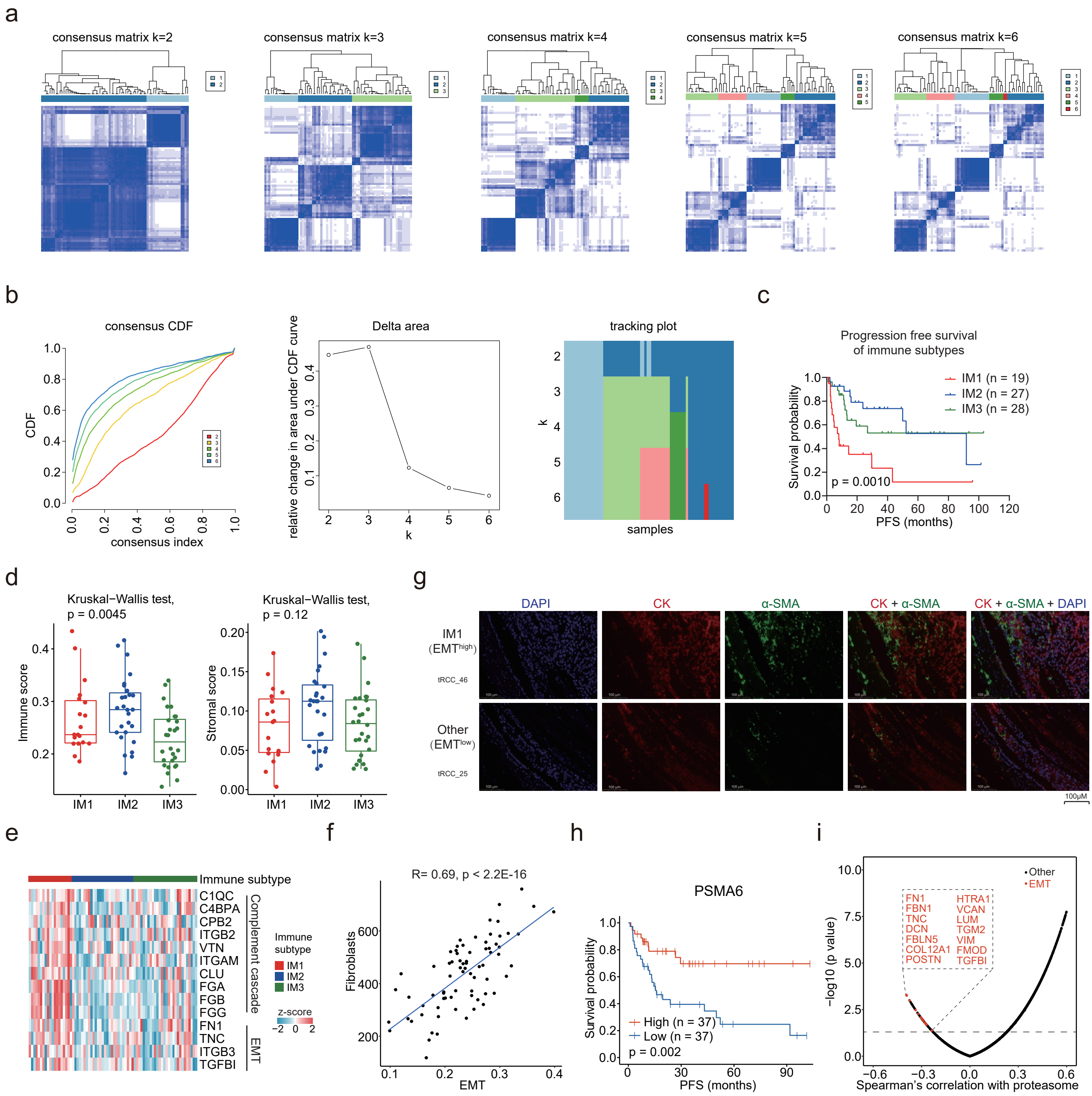

Supplementary Figure 7. Immune Subtyping of tRCC and Proteomic Signatures of Metastatic tRCC

a, Consensus matrices of the tRCC xCell immune signatures, from k = 2 to k = 6. **b,** Cumulative distribution function plot, delta plot, and tracking plot corresponding to consensus matrices from k = 2 to k = 6. **c,** Kaplan–Meier curves of PFS for the three immune subtypes (two-sided log-rank test). IM1, n = 19; IM12, n = 27; IM1, n = 28. **d**, Comparison of pathway scores for the Complement Cascade and EMT among different immune subtypes (IM1, n = 19; IM2, n = 27; IM3, n = 28). P values are derived from Kruskal−Wallis test. Boxplots show the median (central line), the 25–75% IQR (box limits), the ±1.5×IQR (whiskers). **e**, Heatmap of proteins involved in the Complement Cascade and EMT pathways in the three immune subtypes. **f**, Correlation of EMT scores and fibroblasts (two-sided Spearman's correlation test). **g**, Representative immunofluorescence staining of a-smooth muscle actin (a-SMA) and pan-cytokeratin (CK) of a IM1 sample and a non-IM1 sample. Scale bars: 100 μm. **h,** Kaplan–Meier curves of PFS for patients with different PSMA6 abundances (two-sided log-rank test). High expression, n = 37; Low expression, n = 37. **i,** Scatter plot showing the correlation of proteasome scores and protein abundances. EMT associated proteins are annotated.
