# Supplementary material for: Proteogenomic characterization of MiT family translocation renal cell carcinoma
Source: Nat Commun. 2022 Dec 5;13:7494. doi: 10.1038/s41467-022-34460-w (PMC9722939; doi:10.1038/s41467-022-34460-w)
Supplement: Supplementary file 3 — Description of Additional Supplementary Files [file 41467_2022_34460_MOESM3_ESM.pdf]

Description of Additional Supplementary Files

File Name: Supplementary Data 1
Description: Clinical characteristics of 86 Chinese tRCC patients and data availability

File Name: Supplementary Data 2
Description: Genetic alterations in tRCC

File Name: Supplementary Data 3
Description: Transcriptome, proteome and phosphoproteome expression matrix of tRCC tumor and NAT samples

File Name: Supplementary Data 4
Description: Differential analysis between tRCC tumor and NAT

File Name: Supplementary Data 5
Description: Clinical and molecular features among different fusion types of tRCC

File Name: Supplementary Data 6
Description: Proteomic subtypes and molecular features

File Name: Supplementary Data 7
Description: Immune subtypes and associated clinical and molecular features
