# Supplementary material for: Proteogenomic characterization of MiT family translocation renal cell carcinoma
Source: Nat Commun. 2022 Dec 5;13:7494. doi: 10.1038/s41467-022-34460-w (PMC9722939; doi:10.1038/s41467-022-34460-w)
Supplement: Supplementary file 11 — Reporting Summary [file 41467_2022_34460_MOESM11_ESM.pdf]

nature portfolio

Corresponding author(s): Chen Ding, Dingwei Ye, Hailiang Zhang, Jian-Yuan Zhao

Last updated by author(s): Oct 25, 2022

Reporting Summary

Nature Portfolio wishes to improve the reproducibility of the work that we publish. This form provides structure for consistency and transparency in reporting. For further information on Nature Portfolio policies, see our Editorial Policies and the Editorial Policy Checklist.

Statistics

For all statistical analyses, confirm that the following items are present in the figure legend, table legend, main text, or Methods section.

n/a	Confirmed	
☐	☒	The exact sample size (*n*) for each experimental group/condition, given as a discrete number and unit of measurement
☐	☒	A statement on whether measurements were taken from distinct samples or whether the same sample was measured repeatedly
☐	☒	The statistical test(s) used AND whether they are one- or two-sided<br>*Only common tests should be described solely by name; describe more complex techniques in the Methods section.*
☐	☒	A description of all covariates tested
☐	☒	A description of any assumptions or corrections, such as tests of normality and adjustment for multiple comparisons
☐	☒	A full description of the statistical parameters including central tendency (e.g. means) or other basic estimates (e.g. regression coefficient) AND variation (e.g. standard deviation) or associated estimates of uncertainty (e.g. confidence intervals)
☐	☒	For null hypothesis testing, the test statistic (e.g. *F*, *t*, *r*) with confidence intervals, effect sizes, degrees of freedom and *P* value noted<br>*Give P values as exact values whenever suitable.*
☒	☐	For Bayesian analysis, information on the choice of priors and Markov chain Monte Carlo settings
☒	☐	For hierarchical and complex designs, identification of the appropriate level for tests and full reporting of outcomes
☐	☒	Estimates of effect sizes (e.g. Cohen's *d*, Pearson's *r*), indicating how they were calculated

Our web collection on statistics for biologists contains articles on many of the points above.

Software and code

Policy information about availability of computer code

Data collection	Proteome and phosphoproteome samples were analysed on a Q Exactive HF-X mass spectrometer (Thermo Fisher Scientific, Rockford, IL, USA) coupled with high-performance liquid chromatography (EASY-nLC 1200 System, Thermo Fisher Scientific).The mass spectrometry data were acquired using the Xcalibur software v2.2 (Thermo Fischer Scientific). Whole exome sequencing (WES) were all performed on the Nextseq CN500 platform (Illumina).

Data analysis

For WES data, paired-end reads in Fastq format were aligned to a reference human genome (UCSC Genome Browser, hg19) using Burrows-Wheeler Aligner. Variant calling was conducted following GATK best practices. Somatic single-nucleotide variations and small insertions and deletions were detected using MuTect2 (GATK v4.1.2.0) and were annotated using ANNOVAR based on UCSC known genes. CNAs were called following somatic CNA best practice, using the CalculateTargetCoverage function in GATK (v4.1.2.0). RNA-seq reads were mapped onto the human reference genome (GRCh38.p13 assembly) by using STAR software (v2.7.7.a). The mapped reads were assembled into transcripts or genes by using StringTie software (v2.1.4) and the genome annotation file (GCF_000001405.39_GRCh38.p13_genomic.gff). The mass spectrometry raw files were processed in Firmiana, searched against human National Center for Biotechnology Information (NCBI) RefSeq protein database (updated on 04-07-2013, 32,015 entries) using Mascot 2.4 (Matrix Science Inc, London, UK). Statistical analyses were realized by R (v3.6.0). Approaches or algorithms used for the proteome data annotation include factoextra (v1.0.6), survival (v3.2-3), survminer (v0.4.8), CancerSubtypes(v1.20.0), ESTIMATE (v1.0.11), GSEA software (v4.0.3), ssGSEA (v2.0), GSVA (v1.34.0), KSEA(v1.0), corrplot (v0.84), DoRothEA (v1.6.0). Gene annotation was performed using online tools ConsensusPathDB. Whole exome sequencing data was analyzed using GATK (v3.8.1.0 & v4.1.2.0), Maftools (v3.10), Sigminer(c2.1.1), GISTIC2.0 and multiOmicsViz (v1.10.0). Standard statistical tests were used to analyze the clinical data, including but not limited to Wilcoxon rank-sum test, Fisher's exact test, Kruskal-Wallis test, Spearman's correlation test, Pearson's correlation test, log-rank test. Unless otherwise specified, all statistical tests were two-sided, and statistical significance was considered when p value < 0.05. To account for multiple-testing, the p values were adjusted using the Benjamini–Hochberg FDR correction. Kaplan–Meier plots (log-rank test) were used to describe survival.

For manuscripts utilizing custom algorithms or software that are central to the research but not yet described in published literature, software must be made available to editors and reviewers. We strongly encourage code deposition in a community repository (e.g. GitHub). See the Nature Portfolio guidelines for submitting code & software for further information.

Data

Policy information about availability of data

All manuscripts must include a data availability statement. This statement should provide the following information, where applicable:

- Accession codes, unique identifiers, or web links for publicly available datasets
- A description of any restrictions on data availability
- For clinical datasets or third party data, please ensure that the statement adheres to our policy

Proteome and phosphoproteome raw datasets have been deposited to the ProteomeXchange Consortium (dataset identifier: PXD035377) via the iProX partner repository (https://www.iprox.cn/) under Project ID: IPX0003336000 (https://www.iprox.cn/page/project.html?id=IPX0003336000). WES and RNA-seq data are available at NODE (The National Omics Data Encyclopedia) under Project ID: OEP002630 (https://www.biosino.org/node/project/detail/OEP002630). WES and RNA-seq data are also deposited in GSA-human (Genome Sequence Archive for human) in NGDC (the National Genomics Data Center) (https://ngdc.cncb.ac.cn/) under the accession: HRA003190 (https://ngdc.cncb.ac.cn/gsa-human/browse/HRA003190) and HRA002855 (https://ngdc.cncb.ac.cn/gsa-human/browse/HRA002855). The raw sequencing data are available under controlled access due to data privacy laws related to patient consent for data sharing and the data should be used for research purposes only. Access can be obtained by approval via their respective DAC (Data Access Committees) in the GSA-human database. According to the guidelines of GSA-human, all non-profit researchers are allowed access to the data and the Principle Investigator of any research group is allowed to apply for Controlled-access of the data. The user can register and login to the GSA database website (https://ngdc.cncb.ac.cn/gsa-human/) and follow the guidance of "Request Data" to request the data step by step [https://ngdc.cncb.ac.cn/gsa-human/document/GSA-Human_Request_Guide_for_Users_us.pdf]. The approximate response time for accession requests is about 2 weeks. The access authority can be obtained for Research Use Only. The user can also contact the corresponding author directly. Once access has been granted, the data will be available to download for 3 months. Source data are provided with this paper. The remaining data are available within the Article, Supplementary Information or Source Data file.

Human research participants

Policy information about studies involving human research participants and Sex and Gender in Research.

Reporting on sex and gender

This cohort was comprised by 33.7% (n =29) males and 66.3% (n = 57) females.

Population characteristics

This cohort was comprised by 33.7% (n =29) males and 66.3% (n = 57) females, with a median age of 34 years. The 49 patients (57.0%) had stage I/II tumors, and 36 patients (41.9%) had stage III/IV tumors. The majority of tRCC cases showed International Society of Urological Pathology (ISUP) grade 2 (n = 36, 41.9%) and grade 3 (n = 40, 46.5%), and the rest cases showed grade 4 (n = 10, 11.6%). Other information was summarized in Supplementary Data 1.

Recruitment

We retrospectively screened all the 3,850 patients who underwent radical or partial nephrectomy for the treatment of renal tumors at Fudan University Shanghai Cancer Center (FUSCC, Shanghai, China), from January 2008 to December 2020, collecting 86 eligible tRCC cases. The retrospective and single-center design of this study also led to several inherent biases, such as selection bias.

Ethics oversight

The study was compliant with the ethical standards of Helsinki Declaration II and was approved by the institutional review board of FUSCC (050432-4-2108*). Written informed consent was obtained from each patient before any study-specific investigation was conducted.

Note that full information on the approval of the study protocol must also be provided in the manuscript.

Field-specific reporting

Please select the one below that is the best fit for your research. If you are not sure, read the appropriate sections before making your selection.

- [x] Life sciences
- [ ] Behavioural & social sciences
- [ ] Ecological, evolutionary & environmental sciences

Life sciences study design

All studies must disclose on these points even when the disclosure is negative.

Sample size	We screened all the 3,850 consecutive patients who underwent radical or partial nephrectomy for the treatment of renal tumors at FUSCC, one of the largest cancer centers in East Asian, from January 2008 to December 2020, collecting 86 eligible tRCC cases. We collected the cases of this rare type of cancer as much as possible.
Data exclusions	Whole exome sequencing was performed using 86 paired samples, except for 2 patients due to low DNA quality.
Replication	All attempts at replication were successful. The sample sizes are indicated in the figure legends.
Randomization	The tRCC samples for multi-omics processing were not randomized, as investigators were blinded to clinical information.
Blinding	For sample processing, PCA, consensus clustering analysis, all investigators were blinded to clinical information (including TNM stage, ISUP grade, and patients outcomes).

Reporting for specific materials, systems and methods

We require information from authors about some types of materials, experimental systems and methods used in many studies. Here, indicate whether each material, system or method listed is relevant to your study. If you are not sure if a list item applies to your research, read the appropriate section before selecting a response.

Materials & experimental systems

n/a	Involved in the study	
☐	☒	Antibodies
☐	☒	Eukaryotic cell lines
☒	☐	Palaeontology and archaeology
☒	☐	Animals and other organisms
☒	☐	Clinical data
☒	☐	Dual use research of concern

Methods

n/a	Involved in the study	
☒	☐	ChIP-seq
☒	☐	Flow cytometry
☒	☐	MRI-based neuroimaging

Antibodies

Antibodies used	1, Anti-DST antibody (Affinity Bioscience, #DF6752, 1: 200 dilution)<br>2, Anti-TBK1 antibody (Affinity Bioscience, #DF7026, 1: 200 dilution)<br>3, Anti-α-SMA antibody (ServiceBio, #GB111364, 1:1000 dilution)<br>4, Anti-pan-Cytokeratin antibody (Abcam, #ab7753, 1:100 dilution)<br>5, Goat Anti-Rabbit IgG H&L (HRP) (Abcam, #ab205718, 1:5000 dilution)
Validation	1, Anti-DST antibody: http://www.affbiotech.com/download/pdf/DF6752<br>2, Anti-TBK1 antibody: http://www.affbiotech.com/download/pdf/DF7026<br>3, Anti-α-SMA antibody: http://shopobs.servicebio.cn/2021/12/15/1639528817903190425.pdf<br>4, Anti-pan-Cytokeratin antibody (https://www.abcam.cn/pan-cytokeratin-antibody-c-11-ab7753.pdf)<br>5, Goat Anti-Rabbit IgG H&L (HRP): https://www.abcam.cn/goat-rabbit-igg-hl-hrp-ab205718.pdf

Eukaryotic cell lines

Policy information about cell lines and Sex and Gender in Research

Cell line source(s)	Human HEK293T (Cat#CRL-11268; RRID: CVCL_QW54)
Authentication	The cell line was validated by Short tandem repeat (STR) profiling at ATCC Facility.
Mycoplasma contamination	The cell line was tested negtaive for mycoplasma contamination.
Commonly misidentified lines<br>(See ICLAC register)	No commonly misidentified cell lines were used.
